# Reduced Graphene Oxide Modified the Interdigitated Chain Electrode for an Insulin Sensor

**DOI:** 10.3390/s16010109

**Published:** 2016-01-15

**Authors:** Ajay Kumar Yagati, Jinsoo Park, Sungbo Cho

**Affiliations:** 1 Department of Biomedical Engineering, Gachon University, 191 Hambakmoe-ro, Yeonsu-gu, Incheon 21936, Korea; yagati@gachon.ac.kr; 2 Gachon Advanced Institute for Health Science & Technology, Gachon University, 155 Get-Pearl-ro, Yeonsu-gu, Incheon 21999, Korea; jspark88@gc.gachon.ac.kr

**Keywords:** graphene oxide, interdigitated electrode, impedance spectroscopy, capacitive biosensor, insulin

## Abstract

Insulin is a key regulator in glucose homeostasis and its deficiency or alternations in the human body causes various types of diabetic disorders. In this paper, we present the development of a reduced graphene oxide (rGO) modified interdigitated chain electrode (ICE) for direct capacitive detection of insulin. The impedance properties of rGO-ICE were characterized by equivalent circuit modeling. After an electrochemical deposition of rGO on ICE, the electrode was modified with self-assembled monolayers and insulin antibodies in order to achieve insulin binding reactions. The impedance spectra and capacitances were measured with respect to the concentrations of insulin and the capacitance change (ΔC) was analyzed to quantify insulin concentration. The antibody immobilized electrode showed an increment of ΔC according to the insulin concentration in human serum ranging from 1 ng/mL to 10 µg/mL. The proposed sensor is feasible for label-free and real-time measuring of the biomarker and for point-of-care diagnosis.

## 1. Introduction

Human insulin protein (5.8 kDa) is composed of 51 amino acids and two peptide chains referred to as A and B chain which are coupled together by disulfide bonds [1]. The deficiency of insulin can lead to diabetes mellitus or hyperglycemia which is related to high blood sugar levels in the human body [2,3]. The world health organization estimates that 9% of adults aged over 18 have diabetes and 90% of people with diabetes have type 2 diabetes [4]. Thus, detection of insulin is necessary for early disease diagnosis and therapy monitoring of diabetes or insulinoma [5]. The level of insulin has been typically measured by radioassay, enzyme-linked immunosorbent assay [6], turbidimetry [7] and chromatography [8]. Recent advances in sensors suggest more accurate, fast, facile and cost effective measurement of biomarkers, which is alternative or complementary to the established methods.

For direct and efficient measurement of biomarkers, electrochemical impedance spectroscopy (EIS) using microelectrode-based biosensors is recommended since it provides a label-free, real-time measurement analysis [9,10]. EIS is used to characterize the electrical properties of the electrode interface by detecting the changes in the electric field distributed on electrode caused by the adsorption or immobilization of molecules [11]. To increase the measurement sensitivity of molecular recognition, nano- or micro-interdigitated electrodes are highly employed since the electric field could be confined close to the electrode surface according to the gap between the interdigitated electrodes [12]. Due to the small separation distance between the interdigitated electrodes, it has an advantage of an increased collection efficiency of analyte ions with a decreased equilibrium time [13]. Furthermore, the signal to noise ratio is determined by the electrode thickness, one of the structural factors which should be considered for increasing the measurement sensitivity [14]. Since the electric field tends to concentrate more on the electrode edges, the impedance measurement is mostly contributed by the analytes positioned on the electrode edges and thus it results in a disequilibrium in the sensing area. To achieve better measurement sensitivity and to increase the binding efficiency of target anlytes, the modification of the electrode surface has been investigated by deposition of the nanoparticle [15,16], nanowire [17], or graphene [18]. Graphene, a 2D nanomaterial which has excellent electronic and mechanical properties, is an ideal candidate for the electrode material. Much efforts have been focussed on the fabrication of the graphene coated electrode to increase the efficiency of the electrode-based biosensor [19]. Reduced graphene oxide (rGO) was deposited on various electrodes such as glassy carbon [20], indium tin oxide [21] or gold and polymers [22] because of its higher sensitivity with a wide range of analyte in electrochemical detection systems [23]. Electrocatalytic activity of rGO originates from its edge defects that can act as mediators and facilitate electron transfer between analytes and the surface of electrodes (edge plane of rGO has much larger specific capacitance, faster electron transfer rate, and stronger electrocatalytic activity in comparison with those of its basal plane). Furthermore, rGO has been deposited on the electrodes as a hybrid material along with many nanoparticles such as Au, Pt or Ag [24].

In this study, we propose an interdigitated chain electrode (ICE) modified by rGO through potentiostatic electrodeposition and its application towards insulin sensor. Due to the chain-shape of the sensing electrodes in ICE, it would be able to decrease the edge effect of the electric field in comparison to the typical rectangular-shaped interdigitated electrode. Further, rGO deposited ICE (rGO-ICE) would provide better analyte-binding efficiency and measurement sensitivity. With a fabricated rGO-ICE, direct capacitive measurement of the electrode surface or non-Faradaic measurement [25] according to the electrode modification, the adsorption of biomolecule and antigen-antibody binding was investigated instead of the indirect measurement of the charge transfer ions, e.g., ferri- or ferrocyanide redox couple. By analyzing the change in capacitance measured with respect to the concentration of insulin in phosphate-buffered saline (PBS), the feasibility of the developed rGO-ICE for insulin sensor was evaluated. 

## 2. Experimental Section

### 2.1. Reagents and Apparatus

Graphene oxide dispersed in H_2_O, 3-aminopropyl triethoxysilane (APTES, 99%), glutaraldehyde (GA, 50% in H_2_O), Human insulin (recombinant, expressed in yeast (proprietary host)), bovine serum albumin (BSA) were purchased from Sigma-Aldrich (St. Louis, MO, USA). Anti-insulin antibodies (goat anti-rabbit IgG-TR) were obtained from Santa Cruz Biotechnology (Dallas, TX, USA). Human serum was obtained under informed consent at Yonsei Severance Hospital (Seoul, Korea). Phosphate-buffered saline (10 mM PBS, 137 mM NaCl, 2.7 mM KCl, 4.3 mM Na_2_HPO_4_ and 1.4 mM KH_2_PO_4_, pH 7.4) and sodium phosphate buffer (1 M NaH_2_PO_4_ and 1 M Na_2_HPO_4_) were obtained from Tech and Innovation (Gangwon, South Korea). Polydimethylsiloxane (PDMS) was purchased from Dowhitech Silicone Co., Ltd. (Goyang-si, Korea). Deionized (DI) water (18.2 MΩ·cm) was obtained from Milli-Q system and used throughout the experiments. All other chemicals were of analytical grade unless otherwise mentioned. The electrochemical deposition of rGO on ICE or EIS on the developed sensor was performed by using a potentiostat (CompactStat, IVIUM, Eindhoven, The Netherlands). The measured impedance spectra were characterized by nonlinear curve fitting to a designed equivalent circuit model through a commercially available ZView software (Scribner Associates Inc., Southern Pines, NC, USA). Scanning electron microscopy (SEM) images of the modified electrode surface were acquired using COXEM (EM30, Daejeon, Korea) operated at 20 kV.

### 2.2. Fabrication of rGO-ICE

**Figure 1:**
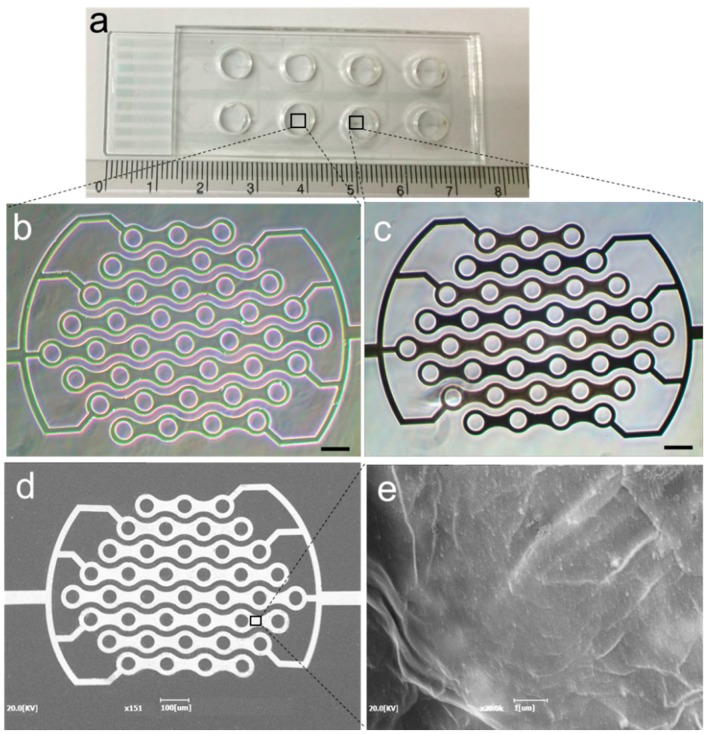
(**a**) Optical images of the fabricated interdigitated chain electrode (ICE) on a glass substrate attached with a polydimethylsiloxane chamber; (**b**,**c**) phase contrast micrographs of the bare and reduced graphene oxide (rGO) deposited on the sensing electrode of ICE respectively; scale bar is 100 µm; (**d**) SEM image of rGO deposited ICE and (**e**) shows the magnified view of the surface structure on one of its electrode arm.

Indium tin oxide (ITO) electrode patterned on a slide glass (75 × 25 × 1 mm) substrate was obtained from Tae Young Optics Co., Ltd. (Incheon, Korea). The patterned ITO electrode consisted of the sensing area, transmission lines and terminal pads. For the insulation of the transmission lines, a low-conductive photoresist (SU-8 2002, Microchem, Newton, MA, USA) was patterned onto the electrode substrate. The width and spacing of the interdigitated fingers were 40 μm and 20 μm, respectively. Then, a polydimethylsiloxane chamber was attached to the electrode chip for electrodeposition of graphene oxide and for preserving liquid during incubation and impedance measurement (Figure 1a). After a solution of graphene oxide (0.5 mM) in phosphate buffer was poured into the chamber, chronoamperometry with a voltage of −1.4 V *versus* Ag/AgCl was applied to the sensing electrodes of ICE for 20 s at room temperature [21]. The rGO deposited ICE (rGO-ICE) was washed with DI water and dried in an N_2_ stream. The micrographs obtained before and after rGO deposition on ICE were shown in Figure 1b,c. Further, SEM images were obtained after electrodeposition of rGO on ICE on both the electrode arms and the surface structure to clearly observe the deposition of rGO layer onto the electrode. The deposited rGO was formed as a wrinkled sheet in Figure 1d,e. 

### 2.3. Development of rGO-ICE Based Insulin Sensor

**Figure 2:**
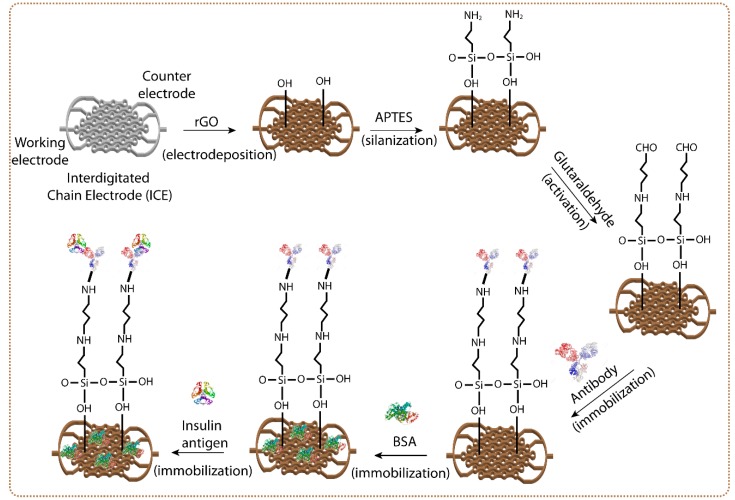
Schematic diagram of the preparation process for rGO-ICE based insulin sensor.

For the immobilization of insulin antibodies on the sensor surface, the sensing electrodes of the fabricated rGO-ICE were immersed with 100 µL of 5% (v/v) APTES in DI water for 3 h at room temperature [26,27]. After washing the electrode with DI water, 50 µL of 1% (v/v) GA in (10 mM PBS, pH 7.4) was drop-cast onto the sensing electrodes of the rGO-ICE and allowed to react with the APTES modified electrodes for 1 h. Afterwards, 10 μL of insulin antibody (IgG) solution (Ab-Ins; 10 μg/mL) was drop cast onto the sensing electrode surface to bind with the APTES/rGO electrode surface [28]. BSA (1 ng/mL) in PBS (10 mM, pH 7.4) was used to block nonspecific adsorption onto the electrode surface. For experiments, different amounts of insulin were mixed into PBS to obtain the insulin concentration of 1, 10, 100, 1000, 5000 or 10,000 ng/mL, and then 10 μL aliquot of the sample was applied to the prepared insulin sensor and incubated at 4 °C for 60 min. The whole process of the electrode modifications and biomolecular immobilizations on rGO-ICE was illustrated in Figure 2. 

## 3. Results and Discussion

### 3.1. Impedance Characteristics of rGO Deposited ICE 

**Figure 3:**
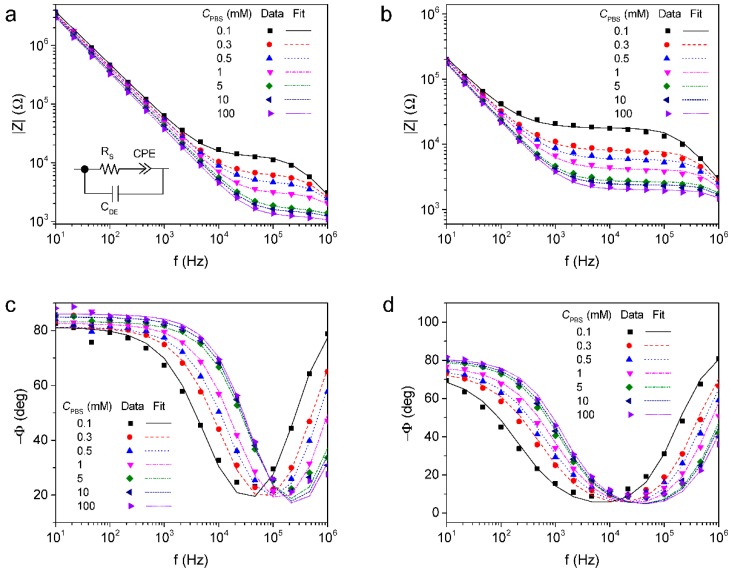
Impedance magnitude of (**a**) bare ICE; (**b**) rGO-ICE and phase of (**c**) bare ICE; (**d**) rGO-ICE measured with respect to the concentration of phosphate-buffered saline (PBS) (Data), and lines fitted using the equivalent circuit model (Fit) consisting of the solution resistance (R_s_), the constant phase element for the electrode interfacial impedance (CPE) and the dielectric capacitance of the solution (C_DE_).

The impedance spectra of the bare or rGO deposited ICE measured in PBS without any redox probes were shown in Figure 3. The impedance magnitude and phase were recorded in a frequency range of 10 Hz to 1 MHz with respect to the concentration of PBS (*C*_PBS_). The impedance characteristic of the electrode was governed by (i) the dielectric capacitance of the solution at high frequencies (C_DE_); (ii) the solution resistance in the mid frequencies (R_S_) and (iii) the constant phase element for the electrode interfacial impedance at low frequencies (CPE). The admittance of the CPE is equal to T(*j*ω)^P^, where T and P are adjustable parameters, *j* is the imaginary unit and ω is the angular frequency (=2πf, f is the frequency) [29]. Table 1 summarizes the extrapolated values of the circuit elements from the fitting results in Figure 3. As the concentration of PBS increased, C_DE_ was increased but R_S_ was decreased. The deposition of rGO led to a decrease in 1/T and P reflecting an increase of surface roughness of overall electrode area, while P for bare ICE was close to 1, indicating that the electrode interfacial impedance was mostly attributed to the capacitive reactance. From the fitting analysis to the measured spectra with PBS, it was found that the impedance characteristic of the fabricated rGO-ICE could be well explained by the designed equivalent circuit model. 

**Table 1:** Extrapolated values of the equivalent circuit elements from the fitting results in Figure 3.

Electrode	*C*_PBS_ (mM)	R_S_ [Ω]	CPE		C_DE_ [×10^−12^ F]
			T [×10^−9^ Ω^−1^ s^P^]	P	
Bare-ICE	0.1	13104 ± 439.5	6.256 ± 0.265	0.901 ± 0.006	53.92 ± 2.205
	0.3	6329 ± 247.85	6.96 ± 0.347	0.900 ± 0.007	55.8 ± 3.46
	0.5	4616 ± 177.07	6.908 ± 0.331	0.901 ± 0.006	56.79 ± 3.879
	1	3038 ± 107.43	6.789 ± 0.298	0.915 ± 0.005	59.31 ± 4.577
	5	1671 ± 43.82	7.070 ± 0.216	0.923 ± 0.004	67.48 ± 5.0678
	10	1481 ± 40.995	6.249 ± 0.20	0.942 ± 0.004	68.78 ± 5.973
	100	1235 ± 47.21	6.267 ± 0.275	0.954 ± 0.005	74.13 ± 9.766
rGO-ICE	0.1	17571 ± 382.02	168.0 ± 7.575	0.812 ± 0.009	53.91 ± 1.833
	0.3	7896 ± 169.28	164.5 ± 6.857	0.824 ± 0.008	56.33 ± 2.316
	0.5	5832 ± 126.34	158.9 ± 6.566	0.833 ± 0.007	58.19 ± 2.660
	1	4106 ± 85.78	151.7 ± 5.974	0.852 ± 0.007	61.0 ± 3.088
	5	2656 ± 41.0	144.1 ± 4.23	0.882 ± 0.005	64.6 ± 3.02
	10	2384 ± 35.4	140.5 ± 3.969	0.890 ± 0.004	66.75 ± 3.138
	100	2003 ± 33.38	140.4 ± 4.389	0.898 ± 0.005	69.5 ± 3.99

### 3.2. EIS Analysis of BSA/Ab-Ins/GA/APTES/rGO-ICE for Insulin Detection

**Figure 4:**
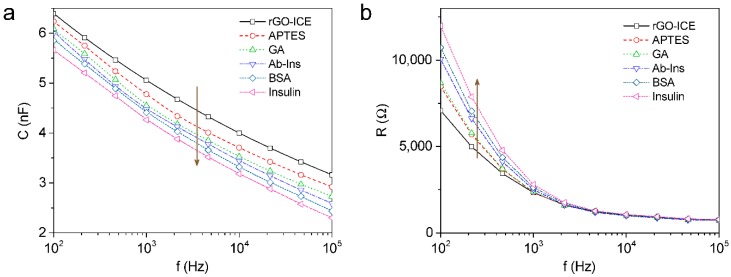
(**a**) Reactive capacitance and (**b**) resistance of the impedance data measured in 10 mM PBS solution (pH 7.0) according to the preparation process for rGO-ICE based insulin sensor.

According to the preparation process for rGO-ICE based insulin sensor, the reactive capacitance was gradually decreased in the frequency range of 100 Hz to 100 kHz but at the same time the resistance below 1 kHz was increased as shown in Figure 4a,b. The decrease in capacitance according to the immobilization of the molecule layer and antigen binding was thought to be caused by the series formation of dielectric layer. The formation of insulating SAM of APTES on rGO-ICE provides a stable dielectric layer on the electrode surface ($C_{\text{SAM}}$). Further binding of insulin antibodies to the electrode results in a new capacitor ($C_{\text{Ab}-\text{Ins}}$) in series with $C_{\text{SAM}}$. BSA acts as a blocking material that fills the empty spaces between the Ab-Ins molecules. Finally, the addition of insulin to the immobilized antibodies forms an additional capacitor ($C_{\text{Insulin}}$) in series.

Therefore, the decrease in capacitance according to the immobilization of the molecular layer and antigen binding can be described as Equation (1) indicating that SAM layer formation or biomolecule immobilization forms a series of capacitive layers. Thus, the total capacitance ($C_{\text{Total}}$) of the rGO-ICE after immobilizing SAM layers, Ab-Ins, BSA and Insulin is given by [25]

(1) $$\frac{1}{C_{\text{Total}}}=\frac{1}{C_{\text{SAM}}}+\frac{1}{C_{\text{Ab}-\text{Ins}}}+\frac{1}{C_{\text{BSA}}}+\frac{1}{C_{\text{Insulin}}}$$

### 3.3. Capacitive Detection of Insulin

**Figure 5:**
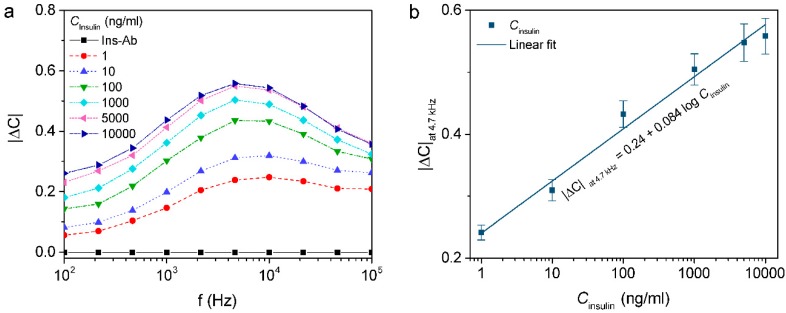
(**a**) Normalized capacitance (|ΔC|) measured with respect to the concentration of insulin diluted in PBS; (**b**) |ΔC|_at 4.7 kHz_ for insulin concentrations ranging from 1 ng/mL to 10 µg/mL.

The change in capacitance (|ΔC|) of the developed rGO-ICE based immunosensor with respect to insulin concentration ranging from 1 to 10,000 ng/mL diluted in 10 mM PBS ($C_{\text{Insulin}}$). With increasing the concentration of insulin, a decrease in capacitance was obtained. The capacitance change *versus* insulin concentration was analyzed by normalization of the capacitance as following,

(2) $$\left|\Delta C\right|=\left|\frac{C_{i}-C_{0}}{C_{0}}\right|$$

where $C_{i}$ and $C_{0}$ are the capacitance measured with and without antigen, respectively. The corresponding |ΔC| *vs.* f was presented in Figure 5a. From the result, it was found that insulin can be detected by using the developed rGO-ICE based insulin sensor, and that |ΔC| could be a parameter for quantitative determination of insulin.

From the results shown in Figure 5a, the maximum change in |ΔC| was observed at f = 4.7 kHz and therefore it was selected for evaluating the sensor performance with respect to insulin interactions. A liner regression curve based on the change in |ΔC|_at 4.7 kHz_ with logarithmic concentrations of antigen ($C_{\text{Insulin}}$) in PBS from 1 ng/mL to 10,000 ng/mL were shown in Figure 5b. The linear regression equation was found to be *y* = 0.084 *x* + 0.240 (*x*: ng/mL, *y*: |ΔC|_at 4.7 kHz_) with R^2^ = 0.985.

### 3.4. Capacitive Detection of Insulin in Human Serum

**Figure 6:**
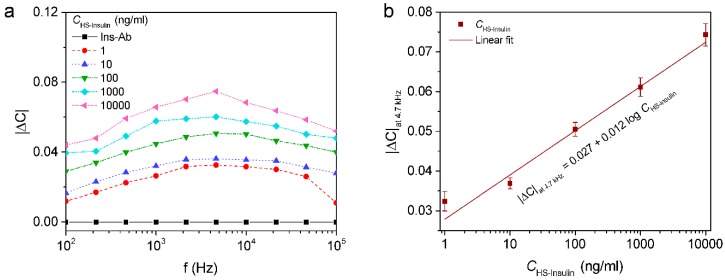
(**a**) Normalized capacitance (|ΔC|) measured with respect to the concentration of insulin diluted in human serum ($C_{\text{HS}-\text{insulin}}$); (**b**) |ΔC|_at 4.7 kHz_ for insulin concentrations ranging from 1 ng/mL to 10 µg/mL.

To achieve the practical applicability, the sensor was subjected to various concentrations of insulin prepared in human serum ranging from 1 to 10,000 ng/mL. The human serum as obtained was diluted in 10 mM PBS buffer (1:200) in order to avoid matrix effects. Various concentrations of insulin in serum diluted samples were prepared and applied to the electrode for incubation. From the measurement of |ΔC| *vs.* f plotted as shown in Figure 6a, it was found that |ΔC| was increased with concentration of the insulin in human serum.

The maximum change in |ΔC| was observed at 4.7 kHz and therefore the |ΔC|_at 4.7 kHz_ was plotted against various concentrations of insulin in serum ($C_{\text{HS}-\text{insulin}}$) shown in logarithmic scale. A linear relationship between the capacitance and logarithmic value of insulin concentrations was found in a range of concentration from 1 to 10^3^ ng/mL with a linear regression equation was found to be *y* = 0.027 *x* + 0.012 (*x*: ng/mL, *y*: |ΔC|_at 4.7 kHz_) with R^2^ = 0.981 and has the detection limit of 0.086 nM which was calculated by 3 SD/S [30]; (where SD is the standard error of the intercept and S is the slope of the calibration curve) by considering the capacitance of the antibody immobilized ICE as a threshold of the signal (Figure 6b). In addition, the detection limit and linear range of the immunosensor were comparable with the other reported insulin sensors as shown in Table 2.

**Table 2:** Comparison of the response characteristics of different modified electrodes for insulin detection.

Electrode	Method	Linear Range (nM)	Detection Limit (nM)	Ref.
SiO_2_NPs-Nafion/GCE ^a^	DPV	10–50	2.8	[31]
NiNPs/ITO	CA	1–125	0.01	[32]
EDA ^b^-CNFs ^c^-NiO	CA	20–1020	12.1	[33]
SPE ^d^/MWCNT ^e^/NiONPs ^f^	CA	20–260	6.1	[34]
CNT-NiCoO_2_/Nafion	CA	17.2–5430	37.93	[35]
GCE/CNT	CV	3.45–68.97	1.34	[36]
Guanine/NiOxNPs/GCE	CA	100–1000	0.022	[37]
Ni(OH)_2_–GN ^g^/GCE	CA	800–6400	200	[38]
rGO/ITO	EIS	0.17–172.4	0.086	This work

^a^ Glassy Carbon Electrode; ^b^ Ethylenediamine; ^c^ Carbon Nanofibers; ^d^ Screen Printed Electrode; ^e^ Multi-walled Carbon nanotube; ^f^ Nickel Oxide Nanoparticles; ^g^ Graphene Nanocomposite.

There was no significant increment in |ΔC| after application of non-target proteins, indicating that the developed sensor showed specificity towards insulin detection. Also, EIS results revealed that the immunosensor has maintained its bioactivity after a storage period of 1 week at 4 °C without any denaturation, indicating that the rGO surface has biocompatibility towards the immobilized biomolecules.

## 4. Conclusions

For label-free and real-time sensing of insulin, a novel design of interdigitated chain electrode with reduced graphene oxide modification was suggested and its feasibility was evaluated from the direct capacitive measurement of the electrode interface. The impedance properties of the reduced graphene oxide deposited electrode were characterized by fitting analysis with an equivalent circuit model. According to the modification of electrode surface towards the insulin sensor, the capacitance change caused by the adsorption of biomolecules could be detected. Further, the capacitance value was decreased with dependency on the concentration of insulin with a detection limit of 0.086 nM. 

As a result, the proposed sensor was considered feasible for label-free and real-time detection of insulin. It is expected that the next stage will be to evaluate the practical applicability of the sensor with clinical human serum samples.
